# Immune Infiltration Landscape in Lung Squamous Cell Carcinoma Implications

**DOI:** 10.1155/2020/5981870

**Published:** 2020-10-10

**Authors:** Jungang Zhao, Wenming Bao, Weiyang Cai

**Affiliations:** ^1^Department of Hepatobiliary Surgery, The First Affiliated Hospital of Wenzhou Medical University, Wenzhou, China; ^2^Department of Gastroenterology, The First Affiliated Hospital of Wenzhou Medical University, Wenzhou, China

## Abstract

Intrinsic cancer cells and the tumor-infiltrating immune cells (TIICs) recruited to the immune microenvironment define the malignant phenotype of lung squamous cell carcinoma (LUSC). Understanding more about the immune microenvironment of LUSC enables the selection of high-risk patients who would derive benefit from immunotherapy. Based on large public LUSC cohorts obtained from TCGA and GEO datasets, 22 types of infiltrating immune cell subgroups were evaluated by CIBERSORT. Meta-analysis, principal component analysis (PCA), single-sample gene set enrichment analysis (ssGSEA), and hierarchical clustering analysis were used to evaluate specific immune responses of LUSC. The distribution of TIICs of LUSC was entirely different from normal. TIIC subpopulations were also found to be closely associated with clinical features and molecular subtypes. Unsupervised clustering analysis revealed that three distinct TIIC subgroups existed with different survival patterns. TIICs are extensively implicated in the pathogenesis and development of LUSC. Characterizing the composition of TIICs influences the metabolism, pathological stage, and survival of tumor patients. It is hoped that this immune landscape could provide a more accurate understanding of the development and immunotherapy of LUSC.

## 1. Introduction

Lung cancer is currently the leading cause of cancer-related death in the world, with high morbidity and mortality, in which non-small-cell lung cancer (NSCLC) accounts for 85% [1]. The main modalities of treatment in use for LUSC currently include surgery, chemotherapy, radiation therapy, molecular targeted therapy, and immunotherapy [2–4]. Notably, despite these options, the overall five-year survival rate of lung cancer was 19%, one of the cancers with poor prognosis [5]. Therefore, it is crucial to uncover effective biomarkers for diagnosis and accelerate development of new classes of antitumor targeted drug.

Immunotherapy, a strategy using immune checkpoint inhibitors to inhibit or activate different immune cell subtypes, can improve the antitumor immune response and get better clinical outcomes. The prognosis of patients receiving immune checkpoint blockers can be significantly improved [6]. Thus, the distribution of TIICs in tumors has been a vital subject for researches. TIICs, consisting of B cells, T cells, macrophages, and other immune cells, are one of the essential components of the tumor microenvironment and play an important role in monitoring antitumor immune responses [7–9]. Drugs targeting specific TIICs have also been found to be associated with better clinical outcomes [10, 11]. Researchers have also revealed the dual role of TIICs in improving body immunity and promoting tumorigenesis, which means that there are complex and undiscovered interactions between TIICs and tumor cells [10, 12].

Newman et al. developed a deconvolution algorithm called CIBERSORT based on gene expression for electronic quantification of various types of immunocytes in heterogeneous samples [13]. CIBERSORT can properly determine the diversity and pattern of TIICs. 22 immune cell types can be enumerated at a time by using CIBERSORT. Because of these, this analysis has received increasing attention in the research of cell heterogeneity [9]. In this research, we used CIBERSORT to assess the proportions of 22 immune cell types in tumor and adjacent samples and analyze their relationship with molecular subpopulations and overall survival. This study is aimed at exploring the intricate relationship between intratumoral immune cell heterogeneity, tumor molecular subtypes, and disease progression in LUSC.

## 2. Materials and Methods

### 2.1. Study Design and Participants

**Figure 1:**
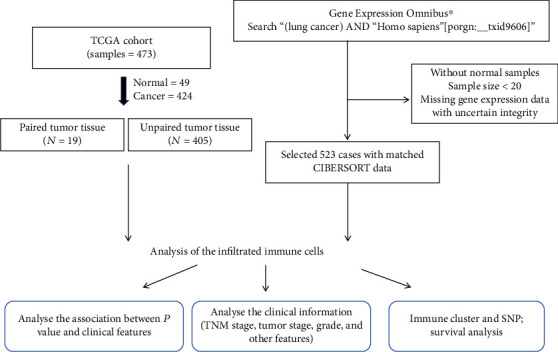
Flowchart detailing the procedure of sample collection and analysis.

The TIIC composition and clinical information used for analysis were obtained from TCGA database up until October 2019. All gene expression data of primary LUSC patients were thought to be qualified, without applying specific criteria for exclusion. Patients with complete information on clinical characteristic, such as gender, age, survival time, and TNM stage, were further analyzed while the rest of the patients were excluded. A total of 424 LUSC expression profiles were included for further study. Table S1 summarizes LUSC patient demographic information. We also screened eligible LUSC microarray in the GEO dataset (http://www.ncbi.nlm.nih.gov/gds/) up until October 2019. All chips with detailed data of gene expression (containing at least 15 samples) from LUSC patients were considered useful, without applying specific criteria for exclusion. A total of 8 chips were included in the further verified study (Table S2): GSE10929, GSE19804, GSE21933, GSE33356, GSE33479, GSE33532, GSE51855, and GSE67061. The flowchart in Figure 1 details the study design.

### 2.2. Evaluation of Tumor-Infiltrating Immune Cells

Taking *P* < 0.05 and [logFC] > 1 as the cutoff criteria, we normalized gene expression data and then identified the differently expressed genes (DEGs) via the limma package. Then, the CIBERSORT algorithm was used to explode the normalized data, which infered the relative ratio of 22 types of infiltrating immune cell. The CIBERSORT algorithm basing on parts of reference gene expression values derives the proportions of various immune cell types from tumor samples mixed with kinds of multiple cells [12]. The CIBERSORT *P* value represents a measure of confidence in the results using Monte Carlo sampling. Immune cell profiles from TCGA and GEO databases were analyzed using CIBERSORT, and the number of permutations was set to 100. The geometric mean of GZMA and PRF1 was calculated to represent immune cytolytic activity [14].

### 2.3. Meta-Analysis

Meta-analysis was manipulated by Review Manager to infer each infiltrating immune cell in LUSC. Continuous outcomes were reported as a standard mean difference (SMD) with a 95% confidence interval (CI). The DerSimonian and Laird method was characterized as the standard method, which offered an average impact estimate of the heterogeneity of effects across a series of chips. We took *P* < 0.05 as statistically significant.

### 2.4. Principal Component Analysis (PCA)

PCA involves a mathematical process in which several potentially related variables are converted into a smaller number of unrelated variables called principal components. The eigenvector associated with the largest eigenvalue has the same path as the first principal component. As a result, the group bias and the individual difference errors have been examined by PCA, thus proving the credibility of the in-depth results.

### 2.5. Single-Sample Gene Set Enrichment Analysis (ssGSEA) and Gene Set Enrichment Analysis (GSEA)

The enrichment scores of the PD-1 immunotherapeutic were calculated by ssGSEA by the gsva package [15, 16]. The PD-1 score was defined as the average of the standardized values of IDO2, TIM-3, IDO2, PDL-1, CTLA4, LAG3, and TIGIT [17]. The correlation between the composition of the TIICs and the immune score was calculated using the Pearson correlation. GSEA based on gene expression profiles was constructed by GSEA.4.0.1. The KEGG pathways significantly altered were identified using cutoff FDR 0.1.

### 2.6. Evaluation of Immunoscore

TMB was defined as the total number of somatic mutations (except silent mutations) and plotted by maftools. According to gene expression profiles, the ESTIMATE algorithm quantifies the immune activity for each tumor sample.

### 2.7. Single Nucleotide Polymorphism (SNP) Analysis

Affymetrix SNP 6.0 arrays were gained by using the VarScan method to analyze the germ cell/somatic cell mutation site data obtained from the second-generation sequencing data of LUSC tissues in TCGA database (https://cancergenome.nih.gov/). We assess the influence of mutant genes on the prognosis of patients with LUSC using the Kaplan-Meier plot and screened out the most frequent mutated genes for further analysis.

### 2.8. Hierarchical Clustering Analysis

Samples were grouped using hierarchical agglomerative clustering, which functioned in assigning a similar sample into a data frame and finding the closest cluster pair. The distribution of samples was shown in the consensus matrix heat map.

### 2.9. Statistical Analyses

The Mann–Whitney *U* test was used for continuous variables. Meta-analysis was conducted by Review Manager 5.3 to verify the proportion of immune cell infiltration, and results were remarked as standard mean difference (SMD) with a 95% confidence interval (CI). The clinical endpoints of this investigation included overall survival (OS) and disease-free survival (PFS). Patients with a CIBERSORT *P* value of ≤0.05 were included in the survival analysis. Log-rank survival analysis was employed to determine the effect of various immune cell infiltrations on patient OS and PFS. Statistical analysis was conducted with SPSS, R language (3.4.4), and GraphPad Prism. Differences among variables were determined to be statistically significant when the *P* value was ≤0.05 (one/two tails).

## 3. Results

### 3.1. Performance of CIBERSORT for Characterizing TIIC Composition of LUSC

**Figure 2:**
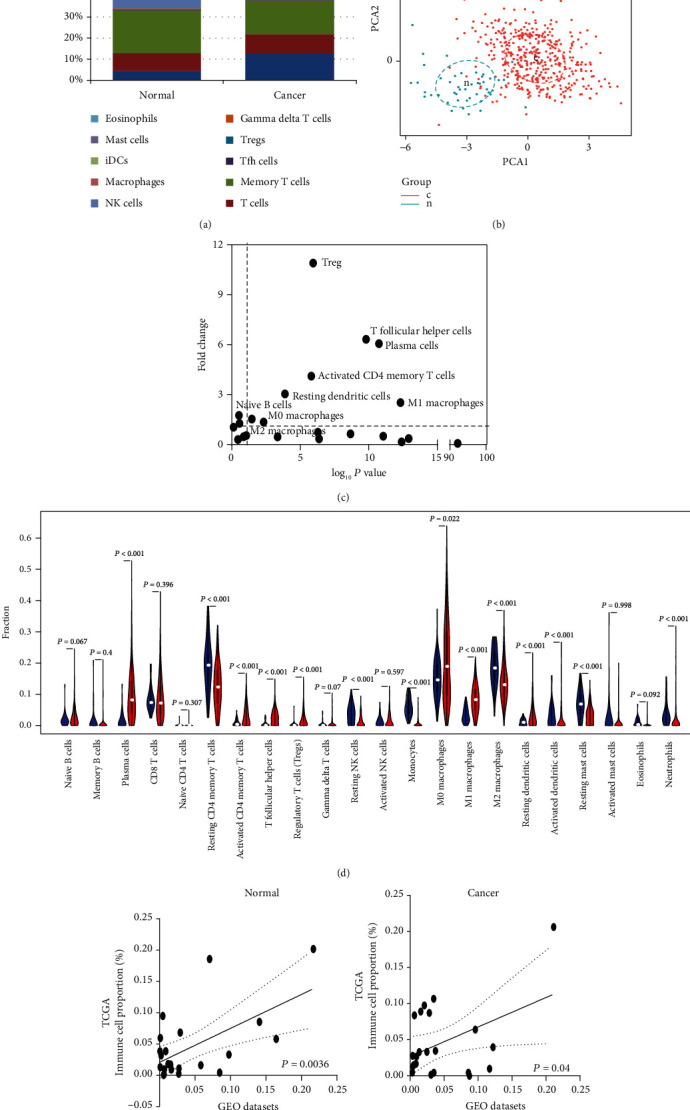
Landscape of microenvironment TIIC composition in LUSC. (a) The composition of TIICs of normal and LUSC tissues. (b) The proportions of TIIC composition from normal and cancer tissues displayed distinct group-bias clustering and individual differences. (c) Volcano plot visualizing the differentially TIIC composition. The point outside the dotted line represents the differential subpopulations with statistical significance (*P* < 0.05) between cancer and normal samples. (d) Violin plot of the proportions of TIIC subpopulation (blue represents normal tissue, red represents LUSC). (e) Relative proportions of 22 TIIC subpopulations are compared between two independent datasets (TCGA and GEO cohort).

CIBERSORT was firstly implemented to calculate the different TIIC subpopulations of LUSC tissue. As shown in Figure 2(a) and sFig 1, the proportion of immune cells in LUSC tissues is quite different from that in normal lung tissues. Meanwhile, the proportions of TIIC composition showed a distinct group-bias clustering and individual difference (Figure 2(b)). We also particularly investigated the TIIC composition between matched cancer and adjacent tissues from 19 patients. As shown in the sFig 2, the majority proportions of immune cells were similar within intergroup. Compared to adjacent tissue, the proportions of plasma, M0 macrophage, and M1 macrophage were higher in LUSC tissues, whereas the fraction of resting CD4 memory T cells and M2 macrophage was relatively lower (Figures 2(c) and 2(d) and sFig 1, *P* < 0.05). On the other hand, we also checked the CIBERSORT outcome in which barcode genes were randomly removed in increments of 10% [18]. As expected, the *P* value was highly sensitive to the reduction of expressed barcode genes (Fig S3). Convincingly, these data did not show cohort bias, which speaks volume for the high reliability of our results.

### 3.2. Meta-Analysis of the Proportions of TIICs in LUSC Tissue

To confirm the accuracy of the above outcome, we further verified its accuracy in other independent LUSC chips both containing tumor and adjacent normal specimens. As shown in Table S2, we downloaded all of the eligible LUSC chips from GEO datasets. Overall, there were 289 LUSC cases and 234 normal tissue samples enrolled in the following exploration. Fig S4 represents the summary of each TIIC composition of the included studies. We merged different platform datasets and eliminated binding batch effect. As shown in Figure 2(e), the proportions of each chip's TIIC subpopulations showed no evident cohort bias to TCGA both in normal and cancer tissues.

**Figure 3:**
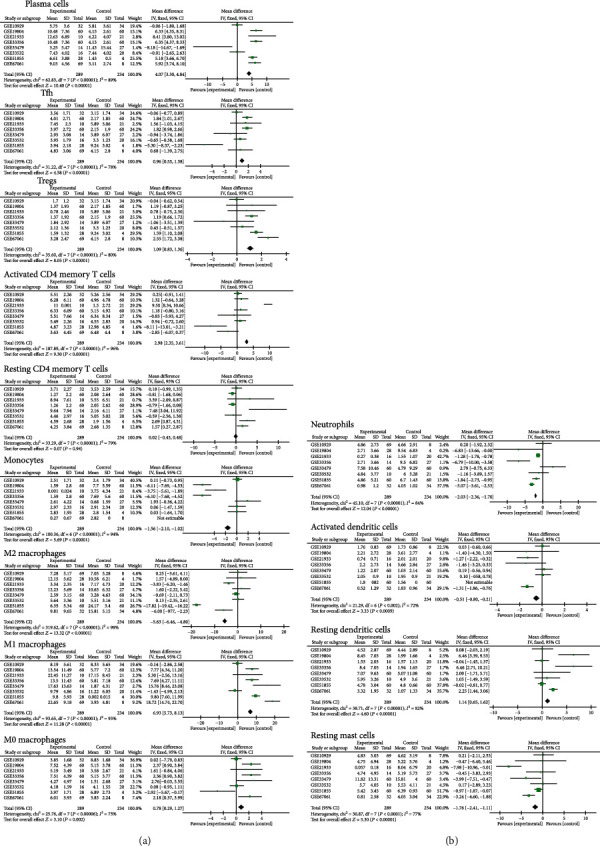
Meta-analysis verified different expression TIIC composition in LUSC.

As is universally acknowledged, meta-analysis is an effective standard way to sum up the results of studies rather than subjective judgment. So, we thoroughly conducted a meta-analysis for all significantly different TIIC compositions. As shown in Figure 3, plasma cells (95% CI, 3.30-4.84; *P* < 0.01), Tfh (95% CI, 0.55 to 1.38; *P* < 0.01), Tregs (95% CI, 0.83 to 1.36; *P* < 0.01), and M1 macrophages (95% CI, 5.73 to 8.13; *P* < 0.01) exhibited an increasing tendency, whereas monocytes (95% CI, -2.10 to -1.02; *P* < 0.01), M2 macrophages (95% CI, -6.46 to -4.80; *P* < 0.01), and resting mast cells (95% CI, -2.41 to -1.11; *P* < 0.01) exhibited downtrends in LUSC tissues in agreement with the previous conclusions (except for resting CD4 memory). Thus, the CIBERSORT results were powerful enough to discriminate TIIC subpopulations in LUSC. Collectively, it highlighted the role of specific TIICs in LUSC, which could provide valuable candidates as diagnostic markers and potential therapy targets for patients.

### 3.3. Proportional Distribution of TIIC Subpopulation and Clinical Characteristics

We further combined clinical characteristics with TIIC composition to investigate whether the TIIC subpopulations were statistically associated with LUSC development. As shown in Figs S5A–S5D, the proportion of activated mast cells, resting mast cells, neutrophils, and T follicular helper cells were correlated with advanced T stage. And the proportions of M1 macrophage and CD8 T cell subpopulation had a strong connection with lymph node metastasis (Figs S5E and S5F). These TIIC subpopulations were also evaluated by univariate Cox regression analysis. As Figs S5G and S5H showed in the forest plot, M2 macrophages (OR: HR = 1.09, *P* = 0.0093), regulatory T cells (OR: HR = 1.53, *P* = 0.029), T follicular helper cells (OR: HR = 1.39, *P* = 0.00862), activated CD4 memory T cells (OR: HR = 1.30, *P* = 0.0011), M0 macrophages (OR: HR = 1.29, *P* = 0.045), and plasma cells (OR: HR = 1.58, *P* = 0.043) were significantly related with poor overall survival, whereas resting mast cells (HR = 0.74, *P* = 0.00049) were correlated with improved OS. Resting mast cells (OR: HR = 0.62, *P* = 0.035) and T follicular helper cells (OR: HR = 1.61, *P* = 0.013) were closely associated with PFS.

### 3.4. Identification of Immune Cluster in LUSC

**Figure 4:**
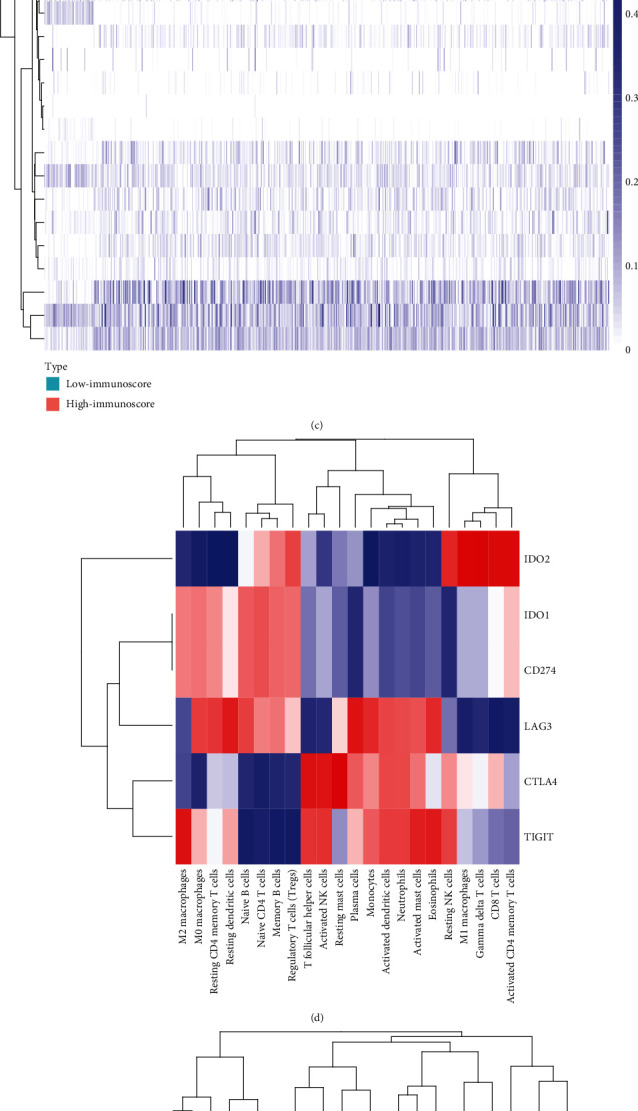
TIIC subpopulation was closely correlated with immune score. (a) CIBERSORT *P* values reflect the overall proportion of immune cells. (b) Spearman correlation matrix of all 22 TIIC compositions. (c) Heat map of the 22 TIIC compositions in high and low immunoscore subgroups. The horizontal axis shows the clustering information of samples which were divided into high and low major clusters. (d) The expression levels of TIIC composition are associated with PD-L1. (e) The expression levels of TIIC composition are associated with APC. (f) The expression levels of TIIC composition are associated with checkpoint.

**Figure 5:**
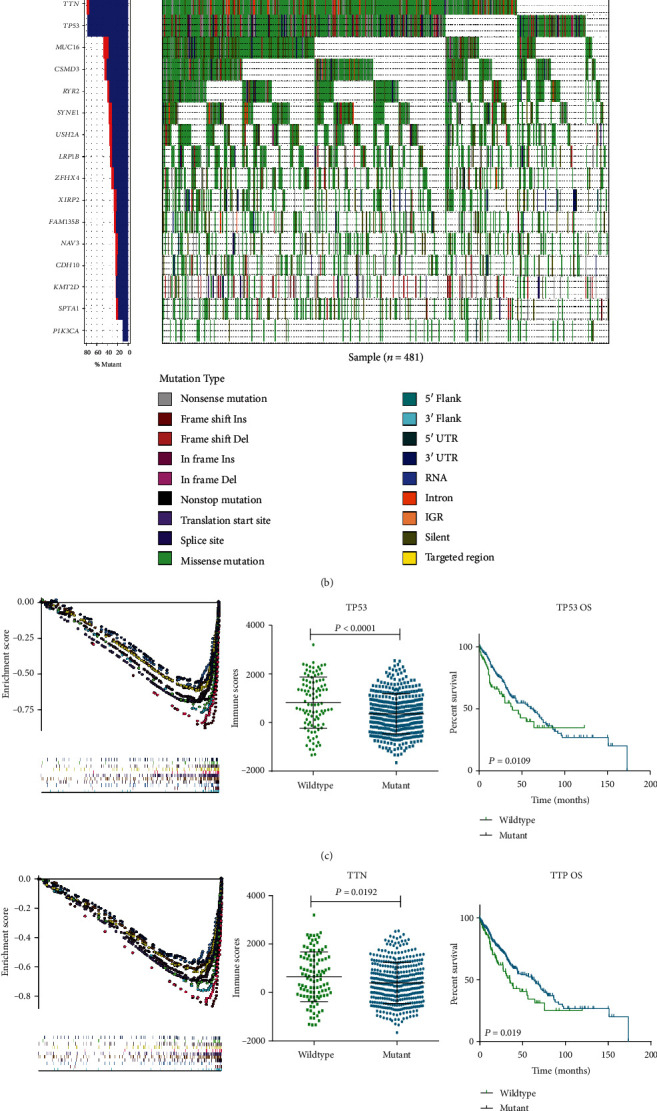
Prognostic associations of subsets of immune cells and molecular subtypes. (a) GSEA differentiates the molecular mechanism of diverse immune score group. (b) Waterfall map depicts the top15 SNPs in TCGA-LUSC. (c, d) GSEA differentiates the molecular mechanism of top 2 SNP wild-type and mutant subgroups. Scattergram of the distribution of immune score between wild-type and mutant subgroups. Survival plots of SNP wild-type and mutant subgroups. (e) Subgroup overall survival analyses of TP53 mutant. The prognostic effect of 22 immune cell subsets by TP53 mutant and wild type. (f) Subgroup overall survival analyses of TTP mutant. The prognostic effect of 22 immune cell subsets by TTP mutant and wild type.

The CIBERSORT *P* value reflected the ratio of immune cells and nonimmune cells, and a greater proportion of immune cells would produce a corresponding smaller *P* value. It is well acknowledged that inflammation cytolytic activity could be described by the geometric mean of GZMA and PRF1 expression [14]. Strong relationship existed in *P* value and inflammation cytolytic activity in both the GEO and TCGA datasets (Figures 4(a) and 4(b), Fig S6). Meanwhile, cytolytic activity of inflammation was most strongly related with the proportion of CD8 T cells (*R*^2^ = 0.3274, *P* < 0.0001) and activated memory T cells (*R*^2^ = 0.26, *P* < 0.0001). Based on the ESTIMATE algorithm, we calculated each patient's immune score distributing between 0.68 and 1.14 and divided them into high vs. low immune score groups in Figure 4(c). Then, we particularly studied the relationship between immune cells and specific immune signatures. Figures 4(d)–4(f), respectively, depict the relationship between immune cells and PD-L1, APC, and checkpoint. It was worth mentioning that the high immune fractions were notably enriched in the T cell receptor signaling pathway, B cell receptor signaling pathway, cytokine interaction, and chemokine signaling pathway(Figure 5(a)).

### 3.5. Immune Cells Associated with Prognosis and Molecular Subtypes

Single nucleotide polymorphisms (SNPs) most frequently occurred in genomic mutations. Then, we firstly analyzed the most frequent SNP mutation in LUSC. As shown in Figure 5(b), TTN and TP53 (respectively, accounting for 81.08% and 80.87%) were the most characteristic SNPs in LUSC. TTN and TP53 mutant distinctly influenced the overall survival of LUSC patients (Figures 5(c) and 5(d)). There was a significant difference of TIIC proportion between the mutant and wild-type subgroups. Surprisingly, both TP53 and TTN mutant subgroups were reversely enriched in the T cell receptor signaling pathway, B cell receptor signaling pathway, cytokine interaction, and chemokine signaling pathway, which was opposite to immunoscore (Figures 5(a), 5(c), and 5(d)). Therefore, we made a detailed analysis of TIIC prognostic effect in TP53 and TTN molecular subtypes. As shown in Figures 5(e) and 5(f), resting dendritic cells played an important role both in TP53 (HR = 1.37, *P* = 0.04) and TTP (HR = 1.66, *P* = 0.007) mutant subgroups. Resting CD4 memory T cells were associated with a favorable outcome in the TP53 mutant subgroup (HR = 0.73, *P* = 0.05), whereas they were associated with an unfavorable outcome in the wild-type subgroup (HR = 2.05, *P* = 0.04).

### 3.6. Identification of Immune Cluster in LUSC

**Figure 6:**
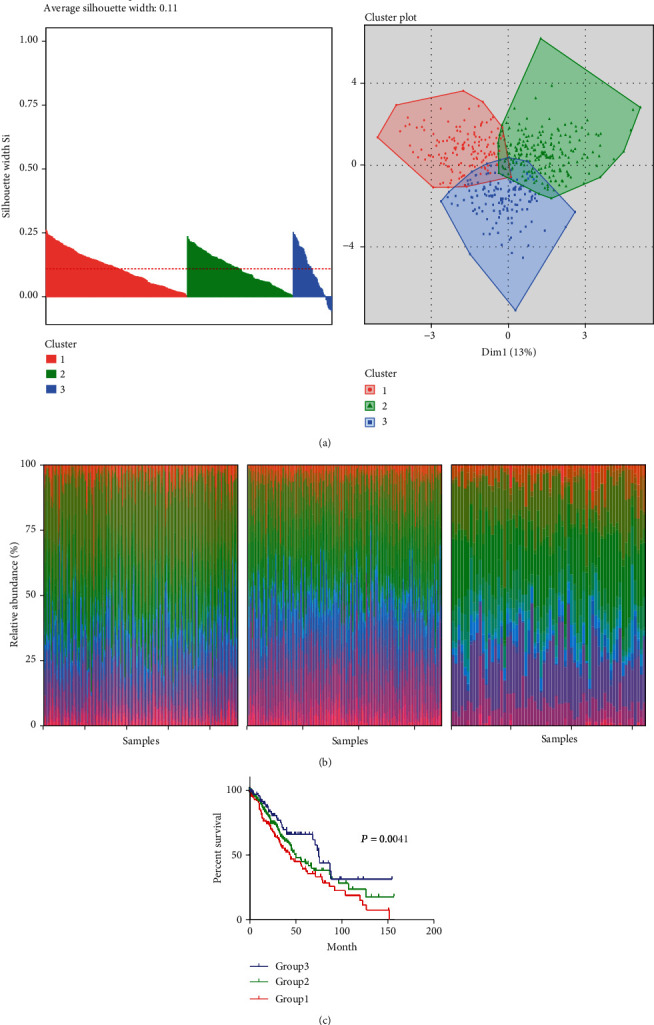
Immune hierarchical clusters associated with LUSC prognosis. (a) Consensus matrix heat map defining 3 clusters of samples. (b) Stacked bar charts of samples ordered by hierarchical cluster. (c) Kaplan-Meier curves for immune score cluster.

On the basis of our above findings, the change of TIIC subsets could significantly influence tumor progression and affect prognosis. In order to explore whether distinct patterns of immune infiltration can be distinguished, we performed hierarchical clustering analysis of 22 TIIC subpopulations and selected the optimal number of clusters by the elbow method. As shown in Figures 6(a) and 6(b), 3 clusters were identified as individualized clusters. Clusters were correlated with distinct immune patterns, and the survival curve is depicted in Figure 6(c). Cluster 1 was characterized by high levels of CD8 T cells, resting dendritic cells, and M0 macrophages. Cluster 2 was enriched with M1 macrophages and resting dendritic cells; cluster 3 was abundant with Tregs and activated dendritic cells. Collectively, characteristic immune clusters could influence clinical outcome (Figure 6(c)).

## 4. Discussion

Lung cancer is currently the leading cause of cancer-related deaths worldwide with a low 5-year survival rate [5]. The main treatments for pulmonary carcinoma are surgery, molecular targeted therapies, and immunotherapy which gradually emerge in recently years [4]. Researchers now focused on the complexity of the tumor microenvironment for its important role in tumorigenesis and suggested that the types and proportions of TIICs might be associated with cancer prognosis [19]. Thus, it is hope that exploring the underlying mechanisms of the relationship between the immune infiltrating cells and prognosis and diagnosis of lung cancer can contribute to discover more effective treatments.

In this study, CIBERSORT analysis, based on the gene expression profiles and deconvolution, was performed to obtain the proportions of 22 immune infiltrating cells in LUSC tissues and paracancerous tissues rather than the analysis of immunohistochemistry which relied on a single marker to distinguish TIIC subsets. Resting CD4 memory T cells, monocytes, M2 macrophage, resting mast cells, resting NK cells, and neutrophils accounted for higher proportions in LUSC tissues than adjacent tissues, opposite to the proportions of plasma cells, resting dendritic cells, M1 macrophage, regulatory T cells, T follicular helper cells, activated CD4 memory T cells, and activated NK cells. Macrophages are one of the main ingredients in tumor microenvironment, which exist in the center and margin of the tumor [19]. A series of experiments have confirmed that M1 macrophages are involved in antitumorigenesis and inflammatory response, while M2 macrophages have an opposite effect of M1 [20]. The effect on tumorigenesis in opposite directions of M1 macrophages and M2 macrophages in lung cancer was also confirmed in other studies [21]. Analysis with PCA plot also identified significant group-bias clustering and individual differences in the ratio of immune infiltrating cells. To further verify the reliability of the results, a meta-analysis was performed on 289 LUSC tissues and 234 adjacent tissues. Regulatory T cells, T follicular helper cells, activated CD4 memory T cells, and M1 macrophages were not conducive to tumorigenesis, while monocytes, M2 macrophages, and resting mast cells had protumor effects.

T follicular helper cells were at an advantage in its relation with favorable OS and PFS. Resting mast cells were bound up with poor OS and PFS. Mast cells are important regulators of the immune response [22]. However, the generalizability of the findings to mast cell inactivation in cancer is still unknown. Oxidized natural polyamines, a kind of tumor-derived secretions, might be the reason leading to the inhibition of mast cells. Natural spermidine and spermidine, oxidized by polyamine oxidase, can prevent IgE from living in vitro [25] and the level of polyamine was high in malignant cells [23, 24]. T follicular helper cells can express Bcl-6, IL-21, and CD40L signals to facilitate the proliferation and differentiation of B cells [25]. Research findings highlight the importance of immune checkpoint therapy to induce T follicular helper cell to activate B cells and inhibit tumor development [26]. Moreover, Tfh may be useful to develop or support ELSs, which can recruit CD8+ T cells, NK cells, and macrophages to participate in antitumor immune responses [27]. Therefore, our findings raise the interesting possibility that T follicular helper cells and resting mast cells might be potential as biological markers in survival prognosis and diagnosis in LUSC patients.

Obvious enrichment of TTN and TP53 mutations in the T cell receptor signaling pathway, B cell receptor signaling pathway, cytokine interaction, and chemokine signaling pathway was observed in LUSC by analyzing mutation points of 481 tissue samples, which was opposite of immunoscore. Resting dendritic cells were positively associated with TP53 mutant and TTP mutant. The TTN mutant and TP53 mutant were noted to have better OS and PFS in contrast to the wild-type TTN and TP53. Resting CD4 memory T cells were negatively associated with TP53 mutation, which in turn had a positive correlation with wild-type TP53. As we know, TP53, a tumor suppressor gene, is the most common mutant gene in many malignancies and the mutation of TP53 is closely relevant to cancer progression, which can be found in around 50% of human cancers [28, 29]. In addition, TP53 mutations also had several strong links to poor prognosis across several cancers such as breast and colorectal [30]. Mutant TP53 has been verified for its ability to promote tumorigenesis, growth, migration, and invasion [31–33]. Most mutations in the coding region are missense mutations (87.9%), while missense mutations account for only about 40% outside of this region and most mutations are nonsense or frameshift mutations [34]. Because mutant TP53 protein accumulates at higher levels in tumors, targeting mutant TP53, including renewing wild-type TP53 activity and depleting mutant TP53, at present shows to be a new therapeutic strategy [35]. Reactivating the resting CD4 memory T cells existing in the lung tumor microenvironment can induce brisker proliferative capacity and secretion of IFN-*γ* to eliminate tumor cells [36]. Blocking CTLA-4 can cause a dramatic expansion of the CTL response to TP53 and the expansion of memory T cells which was closely related to helper T cells [37]. Combined with our conclusions, there may be a corresponding connection between CD4 memory T cells and TP53 in the process of tumorigenesis and development.

In this study, we observed a strong association between the proportions of some TIIC subpopulations and immune cytolytic activity by analyzing the correlation matrix of each proportion and immune cytolytic activity between LUSC tissues and adjacent tissues. To ensure the reliability of the data, the data from TCGA and GEO databases were analyzed, respectively, and the trends of the results are roughly consistent. Moreover, in this study, T follicular helper cells and plasma cells were highly associated with TIM-3 receptors as well as existing high degrees of correlation between regulatory T cells and PDL-1. Programmed cell death protein-1 (PD-1), an important immune checkpoint receptor on the surface of immune cells, plays a pivotal role in regulating immune response; the dislocation and lack of PD-1 can cause autoimmune diseases [38]. PD-L1, one of the ligands of PD-L1, has the ability to help cancer cells to evade the immune system and inhibit the antitumor immune response [39]. Inhibitors against PD-1/PD-L1 have proved to be effective in antitumor response in lung cancer and other tumors [40]. Compared with chemotherapy, pembrolizumab, nivolumab, and atezolizumab, inhibitors of PD-1/PD-L1 immune checkpoint can significantly improve overall survival of NSCLC patients [41–43]. High levels of Tregs are associated with increased tumor infiltration [44]. And the combination of PD-L1 and PD-1 can inhibit T cell receptor-mediated lymphocyte proliferation and cytokine secretion [45]. As described above, it is worth to further explore the relationship between Tregs and PD-1 which might have potential therapeutic value in clinic.

After these, we divided LUSC cancer patients into 3 clusters according to the relative content of 22 TIIC subtypes. The majority of these samples were classified as cluster 1. Moreover, there were classification overlaps among these three clusters. Unlike cluster 1 and cluster 2, cluster 3 conferred better prognosis to patients. Cluster 1 was defined by high levels of CD8 T cells, resting dendritic cells, and M0 macrophages. Cluster 2 was enriched with M1 macrophages and resting dendritic cells, which was consistent with previous associations with survival outcomes. Each cluster has corresponding characteristic function enrichment term. These findings indicate that the character of immune infiltration across lung cancer has considerable variability, which can affect the clinical results.

Nevertheless, this retrospective work still has some limitations. Firstly, the data used for the CIBERSORT analysis was on the basis of TCGA and GEO databases, which lack basic information of patients and contain the unpaired samples. Secondly, though cohort bias had been eliminated by using statistical methods, potential heterogeneity in these data still exists. Thirdly, CIBERSORT can only estimate the relative abundance of immune cells, which means some cell types maybe overestimated or underestimated. Thus, if possible, further experiments would be performed in vivo and in vitro to verify these results and overcome some of these limitations.

In conclusion, we analyzed the proportions of the 22 immune cells in LUSC tissues and adjacent tissues, associated with tumorigenesis. And some specific immune infiltrating cells have the potential for diagnosis and prognosis of LUSC. Moreover, our findings of mutation points are also promising to also contribute to the implementation of immunotherapy and provide the possibility for the development of new immunotherapeutic drugs.

## Data Availability

The data are available in TCGA and GEO datasets.

## Abbreviations

LUSC: Lung squamous cell carcinoma
TIICs: Tumor-infiltrating immune cells
PCA: Principal component analysis
ssGSEA: Single-sample gene set enrichment analysis
NSCLC: Non-small-cell lung cancer
DEGs: Differently expressed genes
SMD: Standard mean difference
CI: Confidence interval
GSEA: Gene set enrichment analysis
OS: Overall survival
PFS: Progression-free survival
SNP: Single nucleotide polymorphism
Tregs: Regulatory T cells.

## Supplementary Materials

Supplementary Materials Supplementary Fig 1: specific TIIC proportion expression in normal and LUCS tissues. Supplementary Fig 2: the composition of TIICs of paired cancer and paracancerous tissue. Supplementary Fig 3: box plot of the distribution of CIBERSORT *P* value and average Pearson's correlation using datasets with progressively fewer (10% increments) barcode genes from the LUSC cohort. The *P* value was highly sensitive to the diminishing representation of the barcode genes. Supplementary Fig 4: landscape of GEO TIIC composition in LUSC. (A) Bar charts summarize the proportion of chips with different *P* value thresholds. (B) Bar charts summarize GEO chip concrete immune cell subset proportions of RCC tissues. Supplementary Fig 5: association between the TIICs and clinicopathological features in LUSC. (A–D) Infiltrating immune cells functioned in distinguishing the T stage. (E, F) Infiltrating immune cells functioned in distinguishing the N stage. (G, H) Forest plots showed the association with each immune cell subset and overall survival and PFS. Supplementary Fig 6: correlation matrix of TIIC proportion expression and inflammation activity. Table S1: the clinical information of TCGA LUSC patients Table S2: the detailed information of GEO chips.
