# Supplementary material for: Immune Infiltration Landscape in Lung Squamous Cell Carcinoma Implications
Source: Biomed Res Int. 2020 Oct 10;2020:5981870. doi: 10.1155/2020/5981870 (PMC7569448; doi:10.1155/2020/5981870)
Supplement: Supplementary Materials — Supplementary Fig 1: specific TIIC proportion expression in normal and LUCS tissues. Supplementary Fig 2: the composition of TIICs of paired cancer and paracancerous tissue. Supplementary Fig 3: box plot of the distribution of CIBERSORT P value and average Pearson's correlation using datasets with progressively fewer (10% increments) barcode genes from the LUSC cohort. The P value was highly sensitive to the diminishing representation of the barcode genes. Supplementary Fig 4: landscape of GEO TIIC composition in LUSC. (A) Bar charts summarize the proportion of chips with different P value thresholds. (B) Bar charts summarize GEO chip concrete immune cell subset proportions of RCC tissues. Supplementary Fig 5: association between the TIICs and clinicopathological features in LUSC. (A–D) Infiltrating immune cells functioned in distinguishing the T stage. (E, F) Infiltrating immune cells functioned in distinguishing the N stage. (G, H) Forest plots showed the association with each immune cell subset and overall survival and PFS. Supplementary Fig 6: correlation matrix of TIIC proportion expression and inflammation activity. Table S1: the clinical information of TCGA LUSC patients Table S2: the detailed information of GEO chips. [file 5981870.f1.docx]

**Supplementary Fig1 specific TIICs proportions expression in normal and lusd tissues.**


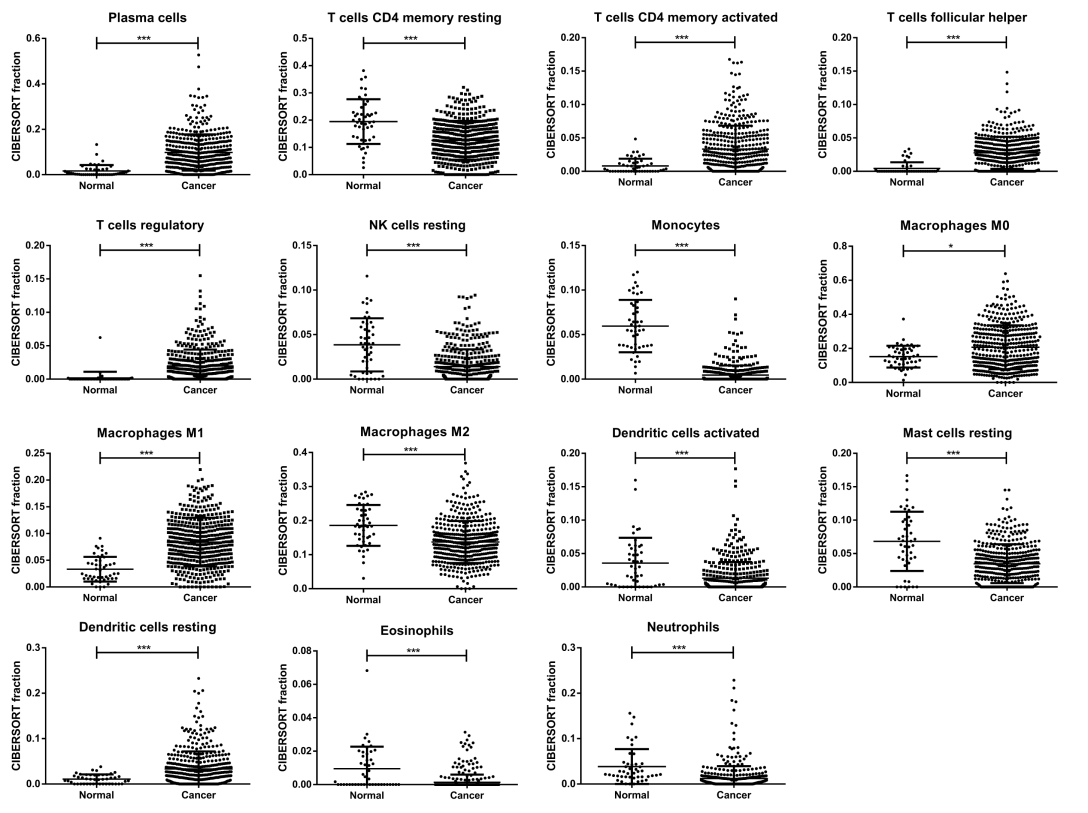


**Supplementary Fig2 The constitute of TIICs of paired cancer and paracancerous tissue.**


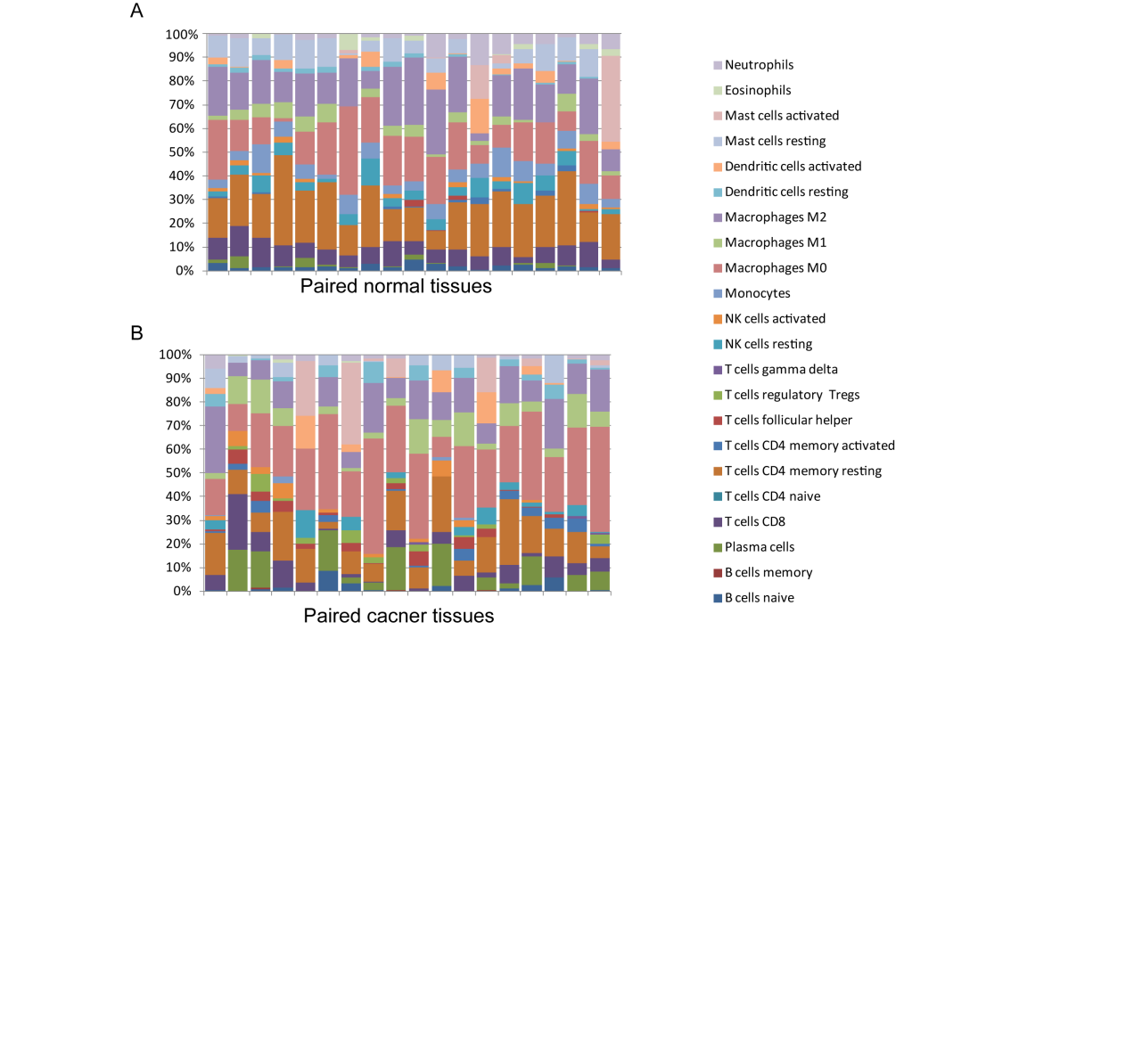


**Supplementary Fig3 Box plot of the distribution of CIBERSORT P-value and average Pearson's correlation using datasets with progressively fewer (10% increments) barcode genes from the LUSD cohort. The P-value was highly sensitive to the diminishing representation of the barcode genes.**


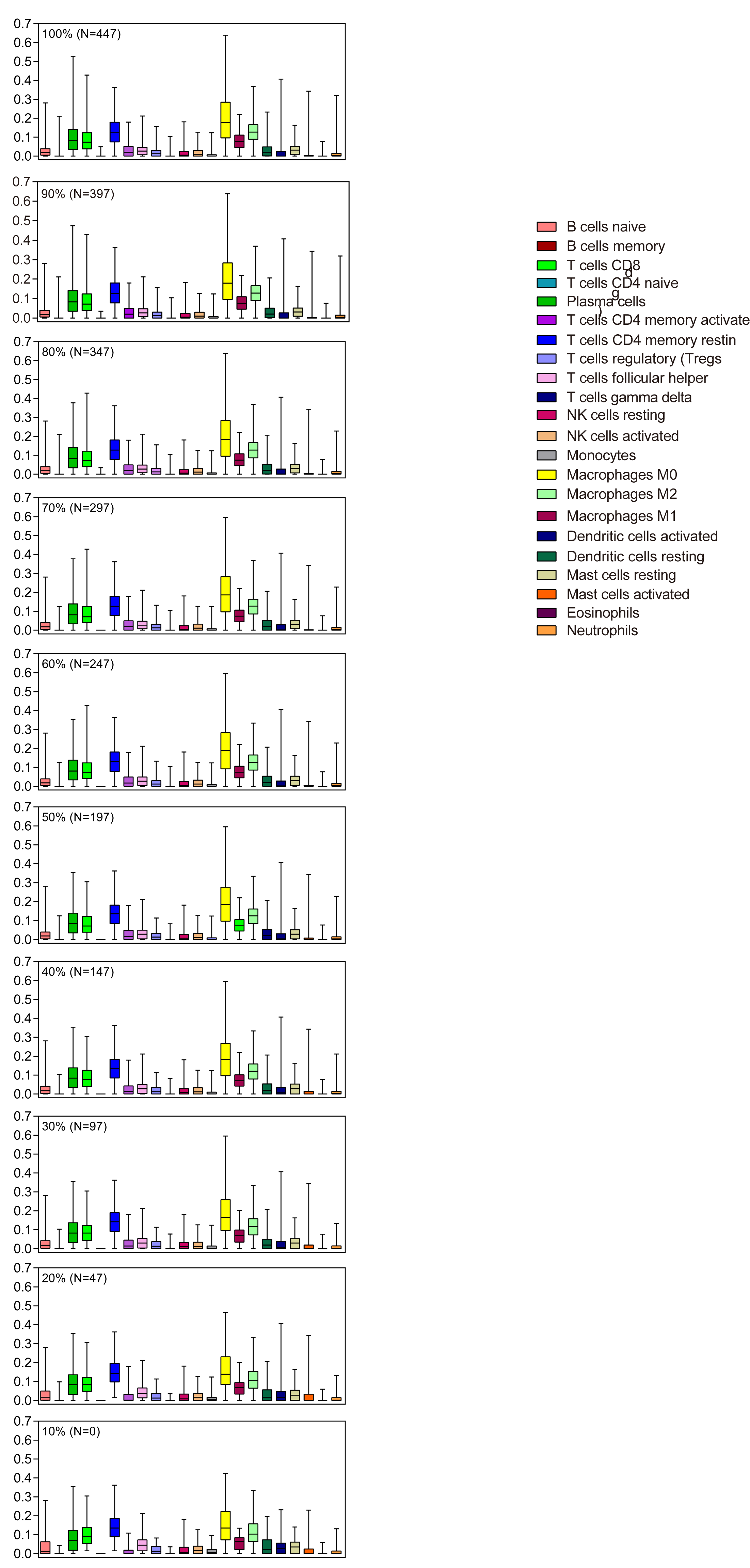


**Supplementary Fig4 Landscape of GEO TIICs composition in lusd. A Bar charts summarise the proportion of chips with different P-value thresholds; B Bar charts summarise GEO chips concrete immune cell subset proportions of RCC tissues.**


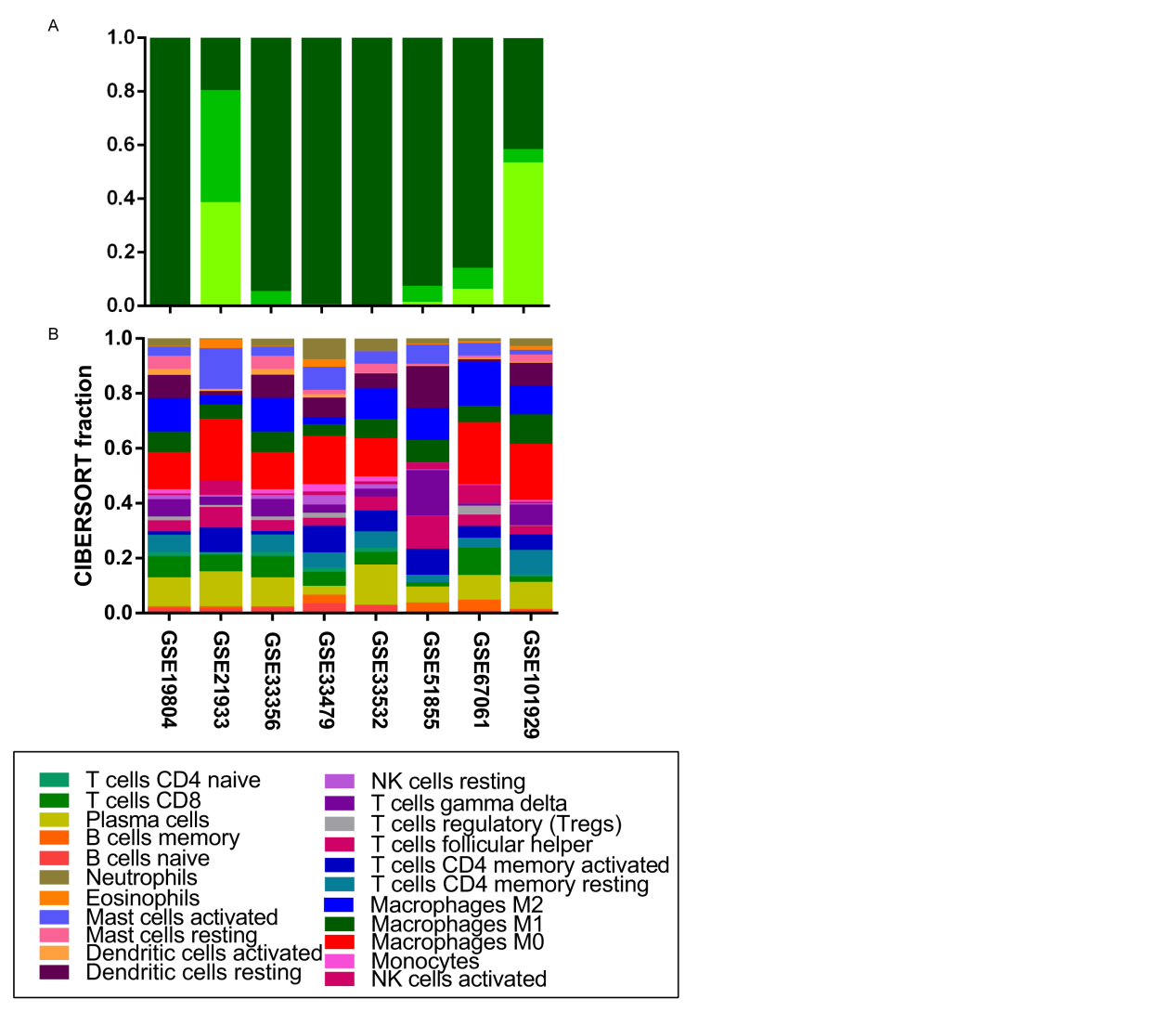


**Supplementary Fig5 Association between the TIICs and clinicopathological features in lusd. (A-D) Infiltrating immune cells functioned in distinguishing the T stage. (E-F) Infiltrating immune cells functioned in distinguishing the N stage; (G-H) Forest plots showed the association with each immune cell subsets and overall survival and PFS.**


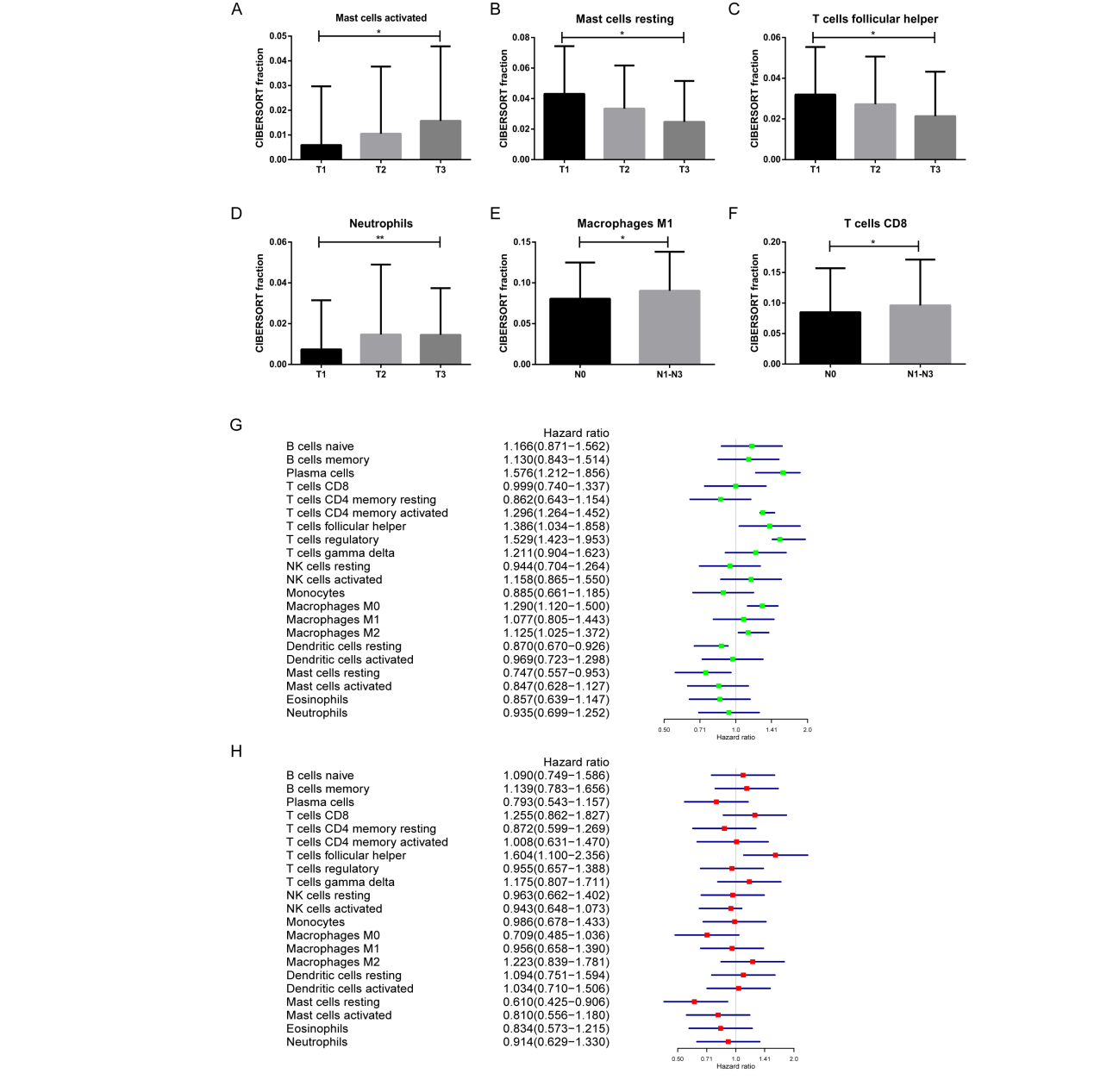


**Supplementary Fig6 Correlation matrix of TIICs proportions expression and inflammation activity.**


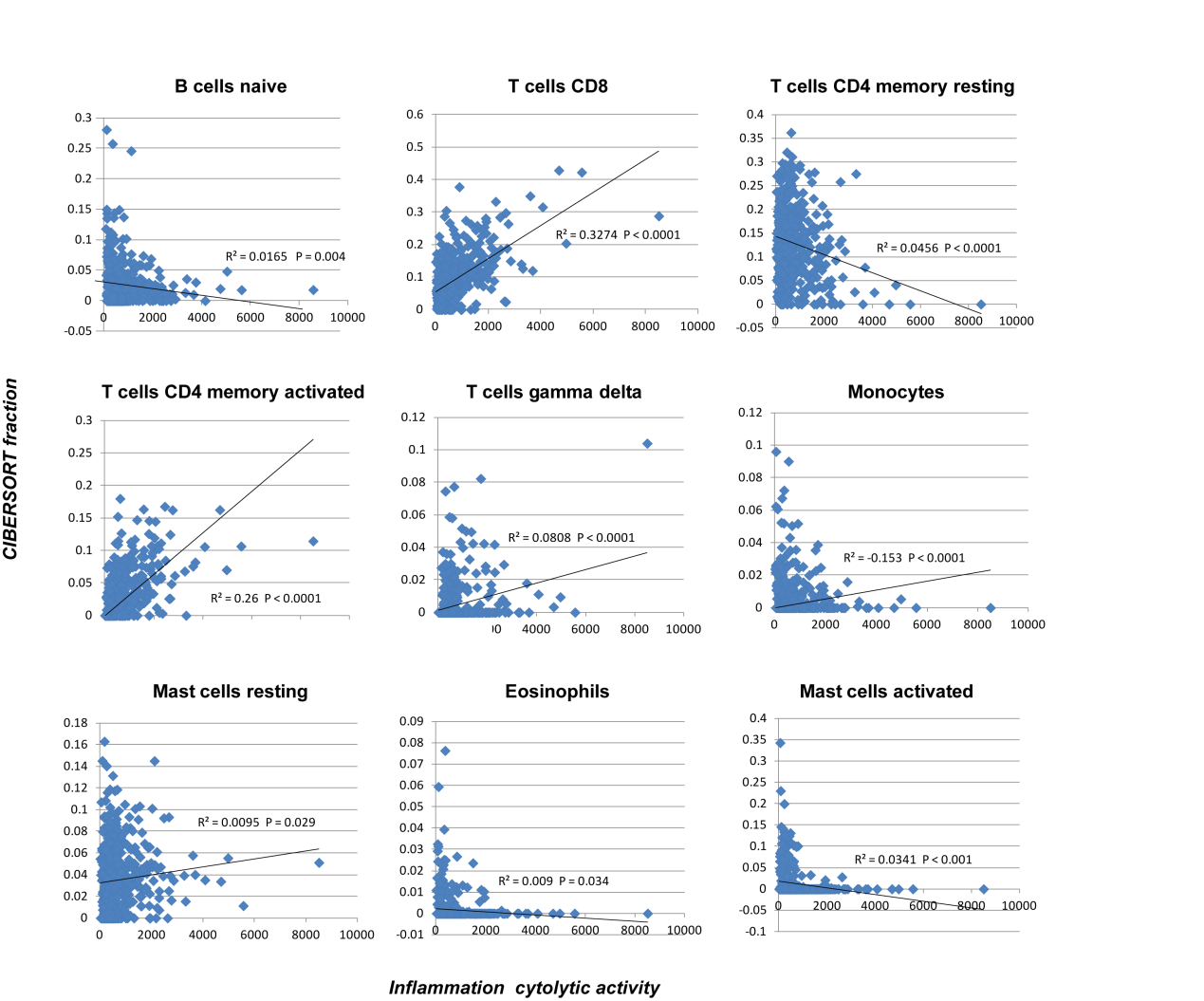


Table S1 The clinical information of TCGA LUSD patients

| Characteristics | N | No. of Patients (%) |
| --- | --- | --- |
| Age (range) | 425 |  |
| Average |  | 67.4 (40-90) |
| < 60 |  | 87 (20.5%) |
| ≥55 |  | 338 (79.5%) |
| Gender | 425 |  |
| Male |  | 311 ( 73.2%) |
| Female |  | 114 ( 26.8%) |
| T stage | 446 |  |
| T1-2 |  | 81 (18.2%) |
| T3-4 |  | 344 (81.8%) |
| Lymph node metastasis | 419 |  |
| N0  N1 |  | 268 ( 64.0%)  112 (26.7%) |
| N2-3 |  | 39 (9.3%) |
| Distant metastasis | 361 |  |
| No |  | 354 ( 92.9%) |
| Yes |  | 7 ( 7.1%) |
| TNM stage | 421 |  |
| Ⅰ-ⅠⅠ |  | 342 ( 84.1%) |
| ⅠⅠⅠ-Ⅳ |  | 78 ( 15.9%) |
| Status | 425 |  |
| Alive |  | 241 (56.7%) |
| Death |  | 184 (43.3%) |
| Follow-un (months) | 423 |  |
| Mean (range) |  | 31.9 (0.03 – 173.7) |

**Table S2 The detailed information of GEO chips**

| Accession | Platform | Number of normal samples | Number of tumor samples |
| --- | --- | --- | --- |
| GSE10929 | GPL80 | 34 | 32 |
| GSE19804 | GPL570 | 60 | 60 |
| GSE21933 | GPL96 | 21 | 10 |
| GSE33356 | GPL96 | 60 | 60 |
| GSE33479 | GPL570 | 27 | 14 |
| GSE33532 | GPL570 | 20 | 16 |
| GSE51855 | GPL15207 | 4 | 28 |
| GSE67061 | GPL6947 | 8 | 69 |
| Total | 523 | 234 | 289 |
